# Early *Babesia canis* transmission in dogs within 24 h and 8 h of infestation with infected pre-activated male *Dermacentor reticulatus* ticks

**DOI:** 10.1186/s13071-018-2637-7

**Published:** 2018-01-17

**Authors:** Marie Varloud, Julian Liebenberg, Josephus Fourie

**Affiliations:** 1 Ceva Santé Animale, 10 Avenue de la Ballastière, 33500 Libourne, France; 2 Clinvet International, P.O. Box 11186, Universitas, Bloemfontein, 9321 South Africa

**Keywords:** Infection, Tick-borne disease, Babesiosis, Piroplasmosis, Companion animals

## Abstract

**Background:**

This study was designed to assess the ability of fed male *Dermacentor reticulatus* ticks to transmit *Babesia canis* to dogs after being detached from previous canine or ovine hosts.

**Methods:**

The study was an exploratory, parallel group design conducted in two trials. All the animals were sero-negative for babesiosis prior to enrolment. In a first trial, donor dogs and donor sheep were infested with *Babesia canis* infected male and uninfected female ticks for 72 h. The ticks were detached and the second group of host dogs were infested for 24 h before tick removal. In a second trial, the experiment was repeated but the donor animals were infested for 88 h and the second group of host dogs were infested for 8 h prior to tick removal. After infestation, the dogs were maintained under clinical surveillance and blood samples were collected for blood smear, IFA and PCR analysis. A dog was considered infected if any of these tests were positive.

**Results:**

All of the dogs (6 out of 6) were infected after being exposed to pre-activated male ticks for 24 h. Half of the dogs were infected after being exposed to pre-activated ticks for 8 h: 1 out of 3 dogs infested with ticks removed from sheep and 2 out of 3 dogs infested with ticks removed from dog. All the infected dogs were positive to blood smear, IFA and PCR. Three of these dogs exhibited elevated body temperature (> 39.4 °C).

**Conclusions:**

This study demonstrates the ability of male *D. reticulatus* to transmit *B. canis* to dogs. The study also illustrates for the first time that, regardless of the first host on which ticks may attach and start feeding, *Babesia canis* can be transmitted to dogs within 8 h of infestation. Since no minimal transmission time can be established for all possible natural situations, a strategy of prevention based on anti-attachment or repellency is recommended.

## Background

*Babesia canis* is a protozoan pathogen infecting red blood cells of canids after being transmitted by ticks. The ornate dog tick, *Dermacentor reticulatus* is the main competent vector tick species of this pathogen and it is widely distributed in Europe [1]. Because of the 48 h minimal duration required for sporogony [2, 3], *Babesia* spp. are generally considered as pathogens with a slow transmission compared to other tick-borne pathogens like Powassan virus, for which a transmission within 15 min of tick attachment in rodents was documented [4]. No minimal transmission time of *Babesia* spp. has been determined so far in dogs. In hamsters, transmission of *B. microti* by *Ixodes dammini* was reported to be more successful after 54 h of attachment than after 36 or 48 h [5]. Consequently, recent models used to assess the efficacy of parasiticides or repellents against the transmission of *Babesia* spp. in dogs allow the adult ticks to feed on the animals for at least 4 days [6]. However, faster transmission of tick-borne pathogens can occur in various situations such as partially-engorged ticks questing [7, 8] or younger life-stages initiating feeding faster [9]. The frequency of these situations is unknown, but tick movements were documented after attachment of *Rhipicephalus sanguineus* between co-housed dogs [10] and partially-engorged questing *Ixodes ricinus* nymphs were observed in the field [11]. For *Ehrlichia canis*, intrastadial transmission by male *R. sanguineus* during multiple feedings on dogs was demonstrated [12]. For *Babesia* spp., immediate transmission after a partial blood meal of moving male *D. reticulatus* ticks has been suggested [13] but never documented. The vectorial competency of male *D. reticulatus* is also unknown. This study was therefore designed to investigate the ability of partially-fed male *D. reticulatus* ticks to transmit *B. canis* to dogs within 24 or 8 h of infestation after being detached from a previous host.

## Methods

**Fig. 1:**
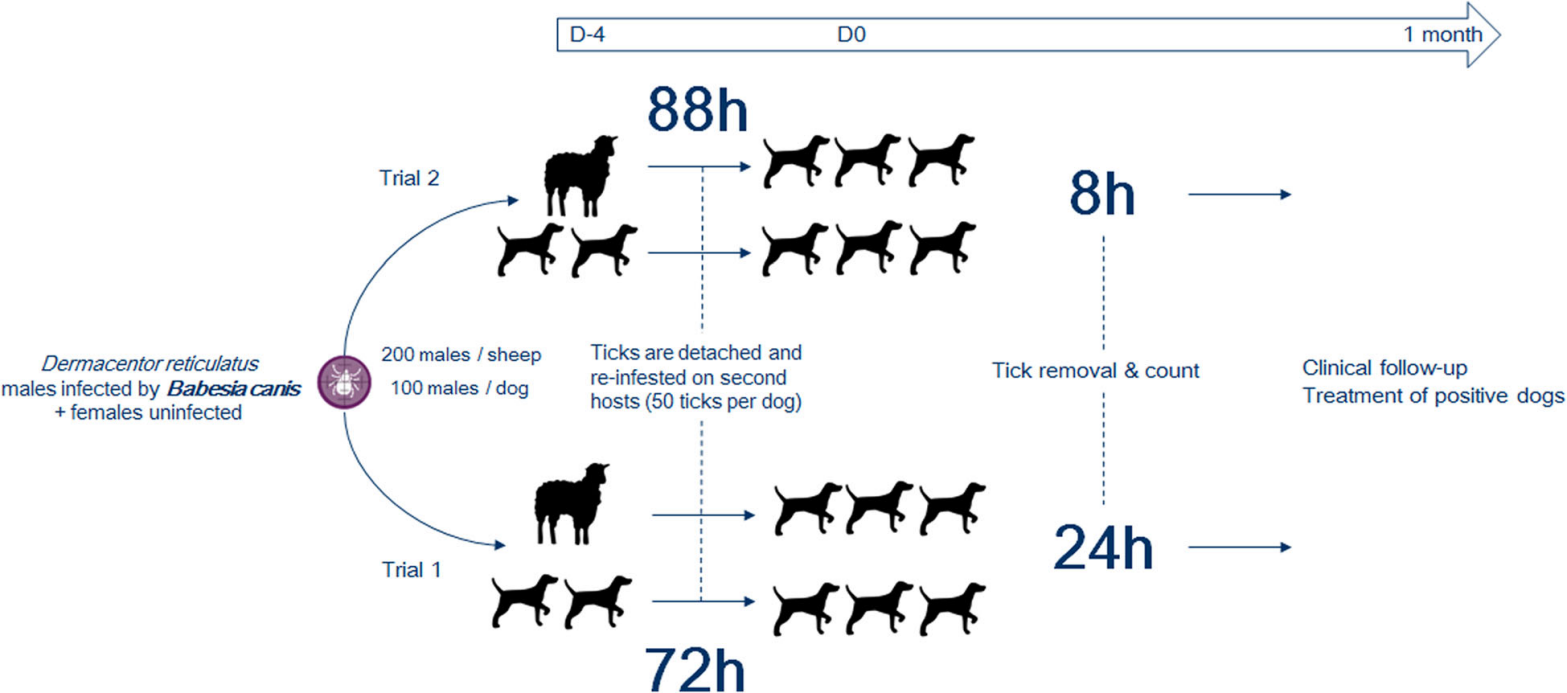
Experimental design

The study was an exploratory, parallel group design, randomized, unicentre trial that was approved by an ethics committee prior to commencement. The study was performed in two sequential steps (experiments 1 and 2) and the design and schedule are described in Fig. 1.

### Animals

Each experiment of the study was conducted on one adult male Merino sheep (group 1, tick host donor) and 3 groups of adult mongrel and Beagle dogs (*n* = 8). All the animals were acclimated to their environment for at least 7 days prior to the start of the study. The sheep were individually identified with a numbered ear tag and were fed hay and a commercial pelleted maintenance concentrate. The sheep were kept individually. The dogs were individually identified by a microchip and were fed commercial dog food once daily. Water was available ad libitum for all the animals. From day -7 to day 8, the dogs were single housed in an indoor kennel. Starting from day 9, the dogs were housed in an outdoor kennel and communally housed within their study groups. All the animals were tested sero-negative for *B. canis* on day -7. In each experiment, the dogs were allocated to groups 2 (*n* = 2, tick donor), 3 (*n* = 3) or 4 (*n* = 3) according to gender and body weight. Clinical examinations were scheduled on dogs on study days -7, 7, 14, 21 and 28. General health observations were performed on a daily basis for the duration of the study.

### Ticks and interrupted feeding

An Irish strain of *D. reticulatus*, enriched with ticks sampled from the Netherlands, was used in this study. All the ticks were adults, 4 months and 5.5 months since the molt, and bred in laboratory conditions. Only the male ticks used for infestation were infected with *B. canis.* These male ticks were taken from a batch of ticks infected with *B. canis* by feeding adult ticks until repletion on a dog with confirmed acute babesiosis. These ticks were subsequently used to propagate a next generation of infected adult ticks. An aliquot of 50 individual male ticks was tested for *B. canis* DNA by PCR to determine the percentage infection.

The sheep (group 1) were infested with 200 (± 16) male *D. reticulatus* infected with *B. canis* and 100 (± 8) female uninfected *D. reticulatus*. The ticks were kept on the sheep in a bag sealed on an area of shaved skin. The dogs (group 2) were infested with 100 (± 8) male *D. reticulatus* infected with *B. canis* and 50 (± 4) female uninfected *D. reticulatus*. The ticks were released on the dogs under light sedation (Domitor®, intramuscular injection at 0.06 ml/kg BW) and maintained in a chamber for up to 1 h after infestation. Tick feeding was interrupted on study day 0, by tick removal with forceps from group 1 and 2 hosts. In experiment 1, tick feeding was interrupted 72 ± 1 h after infestation. In experiment 2, tick feeding was interrupted 88 ± 1 h after infestation. Special care was taken to avoid damage to ticks during removal. At removal from each individual animal, the ticks were counted and categorised as live or dead, attached or free. The sheep from group 1 were euthanized. After tick removal, the dogs from group 2 were treated with diminazine aceturate (Berenil RTU, intramuscular injection of 1.0 ml/20 kg BW) on day 0 and imidocarb dipropionate (Forray 65, subcutaneous injection of 1.2 ml/20 kg BW) against *B. canis* infection.

### Tick challenges

Within 2 h of the tick removal, the dogs from group 3 were infested with ticks taken from sheep (group 1) and the dogs from group 4 were infested with ticks taken from dogs (group 2). Only the male ticks found attached on the groups 1 and 2 animals were used to infest the dogs in groups 3 and 4. Each dog from groups 3 and 4 was exposed individually to 50 ± 3 male ticks infected with *B. canis* and 25 female *D. reticulatus* uninfected ticks on day 0 of the study. The ticks were released on the dogs under light sedation (Domitor®, intramuscular injection at 0.06 ml/kg BW) and maintained in an chamber for up to 1 h after infestation. In experiment 1, tick removal occurred 24 ± 1 h after infestation. In experiment 2, tick removal occurred 8 h ± 15 min after infestation. At removal from each individual animal, the ticks were counted and categorised as live or dead, attached or free.

### Monitoring for babesiosis

Prior to day 0, blood samples were collected for PCR and for serum analysis from dogs in groups 3 and 4. Any abnormal sign reported from daily observations and scheduled veterinary clinical examination led to further inspection of the animals. Rectal body temperature of dogs from groups 3 and 4 was measured at least 3 times per week from day 5 to 28 and at each clinical examination. Animals with abnormally high temperature (> 39.4 °C) or with clinical signs commonly associated with babesiosis were examined and 2 blood smears modified-giemsa stained (Differential Quik Stain Kit, Kyron Laboratories (PTY) Ltd., Johannesburg, South Africa) were assessed for *B. canis* merozoites. If a dog was positive for *B. canis* on a blood smear, approximately 3 ml of whole blood was collected in EDTA tubes for PCR analysis [6] targeting 18S rRNA ITS-1 gene regions [14] prior to treatment. The dogs positive for *B. canis* were immediately treated with diminazine aceturate (Berenil RTU, intramuscular injection of 1.0 ml/20 kg BW) followed 24 h later by imidocarb dipropionate (Forray 65, subcutaneous injection of 1.2 ml/20 kg BW). The dogs were also administered prednisolone 1% (1 ml/10 kg BW id 3 days) and a vitamin mix (Kyro B + Liver, 2 ml sc id 3 days). For serologic analysis, approximately 3 ml of blood sample from all dogs in groups 3 and 4 were collected in dry tubes on days 0 (prior to tick infestation), 14, 21 and 28. During experiment 1, two additional blood samples were collected from 2 dogs (groups 3 and 4) on days 35 and 42. Serum was recovered after centrifugation of blood (25 °C, 3000× *rpm* for 10 min). Serum analysis for *B. canis* antibodies was performed using immunofluorescence antibody assay (IFA, Megaflow® Babesia canis; 1/50 dilution).

### Statistics

Considering the small sample size, no statistical analysis was conducted and the individual data were reported.

### Guidelines

This study was carried in compliance with Good Clinical Practice requirements [15].

## Results

### 24 h transmission (experiment 1)

The BW of dogs on day -7 ranged from 12.7 to 20.3 kg. The proportion of infected male *D. reticulatus* ticks was 16.3% (8 positive ticks out of 49). None of the male ticks (*n* = 206, live attached) were found free or dead on the donor sheep after 72 h of infestation (Table 1). Live male (58–84) and female (21–38) ticks were found successfully attached on donor dogs (group 2). Live male ticks (12–46 per dog) were found attached on all dogs in groups 3 and 4. The average number of live male tick per dog was 33.5 ± 12.9. All the dogs from groups 3 and 4 were positive to all tests for babesiosis (blood smear, PCR and IFAT) and infected by *B. canis* (Table 2). Within 5 to 6 days after being exposed to infected ticks, all the dogs started to exhibit clinical signs typical of babesiosis: enlarged lymph nodes, pale mucous membranes, splenomegaly, panting. The body temperature did not increase above the threshold of 39.4 °C in 4 out of the 6 positive dogs.

**Table 1:** Tick counts and category on animals

Experiment	Group	Animal	Live male free	Live male attached	Live female free	Live female attached	Dead male	Dead female
1	1	CVS2087	0	206	0	90	0	6
	2	86A86D	0	58	0	21	2	0
		B2B7D0	0	84	0	38	0	0
	3	5BBA23	0	34	0	20	10	1
		5D39FA	0	41	0	19	1	0
		E181CA	0	43	0	11	2	0
	4	5E0CAF	0	12	0	13	8	2
		CC1369	0	46	0	20	1	0
		EA19B5	0	25	0	15	16	3
2	1	CVS2540	0	192	3	89	1	1
	2	2892BE	0	63	0	32	6	2
		5C6EF0	0	78	0	30	3	2
	3	2851BE	0	10	0	12	1	0
		5C81F4	0	29	0	7	2	1
		697FD6	0	37	0	13	4	2
	4	1FBF12	0	21	0	11	0	2
		5BF6DC	0	23	0	13	8	4
		B2CA65	0	29	0	14	1	1

**Table 2:** Babesiosis clinical signs and determination

Experiment	Group	Animal	Body temperature (°C, min-max)	Days with temperature > 39.4 °C	Positive blood smear (day)	Positive IFA (day)	Positive PCR (day)
1	3	5BBA23	38.1–40.1	5	5	28	5
		5D39FA	38.0–39.3	None	6	42	6
		E181CA	37.4–38.9	None	6	28	6
	4	5E0CAF	37.8–39.9	5, 23	5	28	5
		CC1369	37.3–38.6	None	6	35, 42	6
		EA19B5	37.5–38.6	None	6	28	6
2	3	2851BE	38.0–38.7	None	–	–	–
		5C81F4	37.2–39.1	None	7	14, 21, 28	7
		697FD6	38.9–39.6	5, 19, 26	–	–	–
	4	1FBF12	37.8–38.6	None	7	14, 21, 28	7
		5BF6DC	37.8–38.4	None	–	–	–
		B2CA65	38.3–38.9	None	7	14, 21, 28	7

### 8 h transmission (experiment 2)

The BW of dogs on day -7 ranged from 12.0 to 21.4 kg. The proportion of infected male *D. reticulatus* ticks was 13.7% (7 positive ticks out of 51). Only one dead male tick and 192 live male attached ticks were found on the donor sheep 88 h after infestation (Table 1). Live male (63–78) and female (30–32) ticks were found successfully attached on donor dogs (group 2). Live male ticks (10–37) were found attached on all dogs in groups 3 and 4. The average number of live male tick per dog was 24.8 ± 9.2. One dog from group 3 and two dogs from group 4 were positive to all tests for babesiosis (blood smear, PCR and IFAT) and therefore infected with *B. canis* (Table 2). Within 7 days after being exposed to infected ticks, these dogs started to exhibit clinical signs such as vomiting, tense abdomen, listless, panting. The body temperature did not increase above the threshold in any of the infected dogs.

## Discussion

### Methodological considerations

Ticks successfully re-attached to dogs after the interrupted feeding on the first hosts and the transmission of the pathogens occurred whatever the host species (dog or sheep) used as a tick host donor. The tick attachment rate of male ticks was numerically higher on sheep hosts (206 and 192 male ticks found attached from about 200 male ticks infested) compared to dogs (58 to 84 male ticks attached for about 100 male ticks infested on dogs). The use of the restrained infestation areas used for sheep vs free infestation on the total body of dogs may have contributed to these differences in attachment rates.

In the present study, male ticks were chosen for infection because they are more likely to move spontaneously between host in natural conditions, as shown for *D. andersoni* [16]. The vectorial competency of the male *D. reticulatus* ticks for *B. canis* was demonstrated prior to these experiments (J. Fourie, personal communication). *Babesia canis*-infected male ticks were already found questing [17]. Male *D. reticulatus* ticks were reported to feed for significant duration and already identified as competent vectors of rickettsial pathogens [18] and *Anaplasma* spp. [19]. Although both sexes can be found on dogs in various proportions [20], the ability of each sex to transmit *B. canis* was not documented.

After the first infestation on sheep or dogs, the female ticks were obviously semi-engorged or engorged with a massive visual enlargement of the aloscutum and changes in coloration. However, the engorgement of male ticks was much less obvious and only visible on the ventral view of the ticks with a moderate increase in the width of the abdomen and a slight change in colour. These observations are in line with previous reports of *D. reticulatus* collected from dogs in natural situations: semi-engorged or engorged female *D. reticulatus* were reported [21] while 48% of the male ticks were categorized as slightly engorged [20]. This lack of visual markers for feeding in male ticks will make them harder to detect by pet owners while the risk of rapid parasite transmission will be increased.

### Transmission times

Despite the moderate percentage infection of ticks (below 20% in both experiments), transmission of the pathogen to the second hosts occurred in both experiments within 24 and 8 h of infestation. No earlier time-points were assessed and it is therefore not possible to determine the actual minimum duration required for transmission, if any. The first infestation obviously provided the necessary stimuli and conditions for the development of *Babesia* into their infective stage within the ticks. Various mechanisms can be involved but are still unknown [22].

These findings are in contradiction with general beliefs about tick-borne disease transmission and related risk. It is indeed most often considered that no transmission can occur for the first 24–48 h after attachment and/or feeding of ticks on their host [23]. For babesiosis, this so-called “grace period” was even longer with 48–72 h of time required after infestation for sporogony of *Babesia canis* in *Dermacentor reticulatus* as observed by microscopy [3] and 36 h *Babesia microti* in *Ixodes dammini*, although no shorter period of transmission was tested [5]. As a consequence, and as demonstrated in experimental situations using strictly unfed ticks [6, 24, 25], veterinarians and pet owners rely on acaricidal products that kill the ticks during this misleading time interval. However, if this concept is suitable to experimental and controlled conditions, it does not simulate real-life situations where the history of vectors is unknown. In natural situations, ticks may detach spontaneously from their host in case of death. They can also be detached by scratching behaviour of the host or detach spontaneously because of mating behaviour. *Rhipicephalus sanguineus* adult ticks were indeed shown to detach spontaneously from dogs [26] and to migrate between dog hosts after attachment [10]. Ticks were also shown to detach from naïve [27] and immunized hosts [28]. As demonstrated for *Borrelia*, the ticks would still be competent vectors, even if they fed on a previously immunized host [29]. The activation of the transmission of bacterial pathogen by interrupted-feeding was already demonstrated with *Rickettsia rickettsii* and nymphs or male *Amblyomma aureolatum* ticks infesting naïve rabbits and guinea pigs. An initial feeding phase of the male ticks of 48 h on rabbits, followed by an immediate transfer on the second host, was able to shorten the transmission time of the pathogen to guinea pigs from 12 h to 10 min [8].

The present experiment did not investigate the minimal transmission time for *B. canis* in dogs. A successful transmission occurred within 8 h of tick infestation but we did not explore shorter time intervals. Since the clock started at the second infestation with ticks being deposited on the ground, close to the dogs, the attachment time of ticks is unknown and expected to vary between individuals. The effect of the time interval between detachment from the first host and re-attachment on the second host is also unknown. In the present experiments, this time interval did not exceed 2 h. However, this time interval is expected to be highly variable in natural situations. No information is available about the maximum time during which the pathogens can be kept alive and in their infective stage. Further experiments should be designed to explore faster transmission of *B. canis* in dogs and the influence of the time interval between detachment from the first host and re-attachment on the second host.

Based on these results, in areas at risk for canine babesiosis or where the vector may spread, we recommend to revise and reinforce the tick-prevention strategy [30]. A multi-modal approach implementing several layers of protection such as reduction of *D. reticulatus* habitat, early removal of any tick found on dogs, prevention of tick attachment by repellent effect and vaccination when available are recommended to prevent the transmission and the development of tick-borne diseases such as babesiosis in dogs.

## Conclusions

These experiments provide evidence of an early transmission of *B. canis* to dogs within 8 h of infestation when the tick vector has a history of interrupted-feeding from a previous host. The study also demonstrates the ability of male *D. reticulatus* to transmit *B. canis* to dogs. In natural conditions, ticks, and in particular males, can detach spontaneously from their host and re-attach. It is impossible to determine the history of individual ticks that may transmit pathogens. Since no minimal transmission time can be established for all possible natural situations, a multi-modal strategy of prevention implementing tick anti-attachment (repellency) is recommended.

## Abbreviations

BW: Body weight
DNA: Deoxyribonucleic acid
EDTA: Ethylenediamine tetraacetic acid
IFA: Immunofluorescent assay
PCR: Polymerase chain reaction
